# The co-transfer of plasmid-borne colistin-resistant genes *mcr-1* and *mcr-3*.*5*, the carbapenemase gene *bla*_NDM-5_ and the 16S methylase gene *rmtB* from *Escherichia coli*

**DOI:** 10.1038/s41598-018-37125-1

**Published:** 2019-01-24

**Authors:** Haiyan Long, Yu Feng, Ke Ma, Lu Liu, Alan McNally, Zhiyong Zong

**Affiliations:** 1 Center of Infectious Diseases, West China Hospital, Sichuan University, Chengdu, China; 2 Division of Infectious Diseases, State Key Laboratory of Biotherapy, Chengdu, China; 3 Department of Infection Control, West China Hospital, Sichuan University, Chengdu, China; 4 Center for Pathogen Research, West China Hospital, Sichuan University, Chengdu, China; 5 Institute of Microbiology and Infection, College of Medical and Dental Sciences, University of Birmingham, Birmingham, UK

## Abstract

We found an unusual *Escherichia coli* strain with resistance to colistin, carbapenem and amikacin from sewage. We therefore characterized the strain and determined the co-transfer of the resistance determinants. Whole genome sequencing was performed using both Illumina HiSeq X10 and MinION sequencers. Short and long reads were subjected to *de novo* hybrid assembly. Sequence type, antimicrobial resistance genes and plasmid replicons were identified from the genome sequences. Phylogenetic analysis of all IncHI2 plasmids carrying *mcr-1* available in GenBank was performed based on core genes. Conjugation experiments were performed. *mcr-3*.*5* was cloned into *E*. *coli* DH5α. The strain belonged to ST410, a type with a global distribution. Two colistin-resistant genes, *mcr-1*.*1* and *mcr-3*.*5*, a carbapenemase gene *bla*_NDM-5_, and a 16S methylase gene *rmtB* were identified on different plasmids of IncHI2(ST3)/IncN, IncP, IncX3 and IncFII, respectively. All of the four plasmids were self-transmissible and *mcr-1*.*1*, *mcr-3*.*5*, *bla*_NDM-5_ and *rmtB* were transferred together. *mcr-1*-carrying IncHI2 plasmids belonged to several sequence types with ST3 and ST4 being predominant. MIC of colistin (4 μg/ml) for DH5α containing *mcr-3*.*5* was identical to that containing the original *mcr-3* variant. In conclusion, carbapenem resistance, colistin resistance and high-level aminoglycoside resistance can be transferred together even when their encoding genes are not located on the same plasmid. The co-transfer of multiple clinically-important antimicrobial resistance represents a particular challenge for clinical treatment and infection control in healthcare settings. Isolates with resistance to both carbapenem and colistin are not restricted to a given sequence type but rather are diverse in clonal background, which warrants further surveillance. The amino acid substitutions of MCR-3.5 have not altered its activity against colistin.

## Introduction

Colistin is the last resort antimicrobial agent to treat infections caused by most Gram-negative bacteria commonly seen in clinical settings, including *Escherichia coli*, *Klebsiella pneumoniae*, *Acinetobacter baumannii* and *Pseudomonas aeruginosa*^1,2^. Bacterial strains that have acquired resistance to colistin have emerged worldwide. In addition to mutations or interruptions in certain chromosomal genes, acquired resistance to colistin has occurred due to plasmid-borne genes^1^. Eight plasmid-borne colistin resistance genes, i.e. *mcr-1*^3^, *mcr-2*^4^, *mcr-3*^5^, *mcr-4*^6^, *mcr-5*^7^, *mcr-6*^8^, *mcr-7*^9^ and *mcr-8*^10^, have been reported. The co-existence of two plasmid-borne colistin-resistant genes in bacterial isolates is uncommon, but recently, we reported the co-existence of *mcr-1* and *mcr-3* plus the carbapenemase gene *bla*_NDM-5_ in an *E*. *coli* clinical strain, WCHEC020123, of phylogenetic group A and sequence type 206 (ST206)^11^. Here we report a second independent occurrence of the co-existence of *mcr-1*, *mcr-3* and *bla*_NDM-5_ in another *E*. *coli* strain, which was recovered from hospital sewage, belonging to a different sequence type (ST410). This strain has an even broader antimicrobial resistance spectrum than the extensive drug resistant WCHEC020123.

## Materials and Methods

### Recovery of the strain and *in vitro* antimicrobial susceptibility testing

*E*. *coli* strain WCHEC025943 was recovered from the influx mainstream of hospital sewage at West China Hospital, Chengdu, western China, in April 2017. The sewage sample was mixed with 100 ml brain heart infusion broth (Oxoid, Hampshire, UK) in a 500 ml flask. After overnight incubation at 37 °C, the culture suspension was diluted to 0.5 McFarland standard and an 100 μl aliquot was plated onto a CHROMAgar Orientation agar plate (CHROMAgar, Paris, France) containing 4 μg/ml colistin and 16 μg/ml meropenem. The plate was then incubated at 37 °C overnight. The pink colony that represents *E*. *coli* was screened for *mcr-1* as described previously^3^. Species identification was established by Vitek II (bioMérieux, Marcy-l′Étoile, France) and by MALDI-TOF MS (Bruker, Billerica, MA, USA).

MICs of amikacin, aztreonam, aztreonam-avibactam, ceftazidime, ceftazidime-avibactam, ciprofloxacin, colistin, imipenem, meropenem, tigecycline and trimethoprim-sulfamethoxazole were determined using the broth microdilution method of the Clinical and Laboratory Standards Institute (CLSI)^12^. For ceftazidime-avibactam, colistin and tigecycline, the breakpoints defined by the European Committee on Antimicrobial Susceptibility Testing (EUCAST) (http://www.eucast.org/) were used, while the breakpoints of aztreonam were applied for aztreonam-avibactam.

### Whole genome sequencing and analysis

Genomic DNA of strain WCHEC025943 was prepared using the QIAamp DNA Mini Kit (Qiagen, Hilden, Germany) and was subjected to whole genome sequencing using both the HiSeq X10 platform (Illumina, San Diego, CA, USA) and the long-read MinION Sequencer (Nanopore, Oxford, UK). The *de novo* hybrid assembly of both short Illumina reads and long MinION reads was performed using Unicycler^13^ under conservative mode for increased accuracy. Complete circular contigs generated were then corrected using Plion^14^ with Illumina reads for several rounds until no change was detected.

Sequence type was determined using the genome sequence to query the *E*. *coli* multi-locus sequence typing database (http://enterobase.warwick.ac.uk/species/index/ecoli). Antimicrobial resistance genes were identified from genome sequences using ResFinder at https://cge.cbs.dtu.dk/services/ResFinder/. Plasmid replicon types and sequence types of IncHI2 and IncF plasmids were determined using PlasmidFinder and pMLST tools at https://cge.cbs.dtu.dk/services/PlasmidFinder/ and https://cge.cbs.dtu.dk/services/pMLST/. Single nucleotide polymorphisms (SNPs) between strain WCHEC025943 and strain WCHEC14828 (also called WCHEC005828, GenBank accession no. RIAW00000000), a *bla*_OXA-181_-carrying ST410 *E*. *coli* identified in the same hospital in 2014^15^, was determined from a two-way whole genome alignment in HarvestTools^16^.

### Nucleotide sequence accession numbers

Complete sequences of the chromosome and plasmids of strain WCHEC025943 have been deposited into GenBank under the accession no. CP027199 to CP027205.

### Phylogenetic group typing

*E*. *coli* phylogenetic group of strain WCHEC025943 was determined using PCR as described previously^17^.

### Cloning of *mcr-3*.*5*

The complete coding sequence of *mcr-3*.*5* was amplified with primers mcr3.5-up (CTGGTCGGAGATATGGGTGT) and mcr3.5-dw (GGCATTCAACATCAGAGCAA) using PrimeSTAR Max DNA Polymerase (Takara, Dalian, China). The primers were designed to amplify the gene with 222-bp upstream and 540-bp downstream sequences of *mcr-3*.*5*. Amplicons were ligated to the pMD20-T vector using the Mighty TA-cloning kit (Takara). The ligated fragments were transformed into *E*. *coli* DH5α. *mcr-3*.*5-*containing transformants were selected on LB agar plates containing 2 μg/mL colistin. The presence of *mcr-3*.*5* in transformants was confirmed by PCR. MIC of colistin was determined for transformants carrying *mcr-3*.*5* using the broth microdilution method^12^.

### Phylogenetic analysis of IncHI2 plasmids

Complete sequences of all IncHI2 plasmids carrying *mcr-1* (n = 25 in addition to pMCR1_025943 and pMCR1_020123) were retrieved from GenBank. Plasmid replicon types and sequence types of these plasmids were determined using PlasmidFinder and pMLST. Annotation was performed using Prokka^18^ and antimicrobial resistance genes were identified using ResFinder. Orthologues of these plasmids were identified using OrthoFinder^19^ with default settings, resulting in a sum of 56 genes representing the core genome of these 27 plasmids. The alleles of orthologous genes were aligned using MAFFT^20^ and concatenated into a single sequence containing 56 aligned genes for each plasmid. The maximum-likelihood phylogenetic tree was inferred based on the core genome using RAxML^21^ with a 1000-bootstrap test.

### Conjugation

Conjugation experiments were carried out in brain heart infusion broth at 30 °C using azide-resistant *E*. *coli* strain J53 as the recipient. Transconjugants were selected on LB agar plates containing 150 μg/ml sodium azide plus 2 μg/ml colistin for *mcr-1*.*1* and *mcr-3*.*5*, plus 1 μg/ml meropenem for *bla*_NDM-5_ or plus 64 μg/ml amikacin for *rmtB*. Transconjugants were also selected on LB agar plates containing 150 μg/ml sodium azide plus 2 μg/ml colistin, 1 μg/ml meropenem and 64 μg/ml amikacin to examine whether *mcr*, *bla*_NDM-5_ and *rmtB* could be transferred together. The presence of *mcr-1*.*1*, *mcr-3*.*5*, *bla*_NDM-5_ and/or *rmtB* in transconjugants was screened using PCR and Sanger sequencing. Conjugation frequency was calculated as the number of transconjugants per recipient cell.

## Results and Discussion

Strain WCHEC025943 was recovered from the sewage sample and grew on the agar plate containing 4 μg/ml colistin and 16 μg/ml meropenem. The complete genome sequence of strain WCHEC025943 was obtained, which was 5.1 Mb and contained a 4.82 Mb circular chromosome and six plasmids of different replicon types (Table 1).

**Table 1:** Plasmids and antimicrobial resistance genes in strain WCHEC025943^1^.

Plasmid	Replicon type	Size (bp)	Antimicrobial resistance genes				
			Colistin resistance	Carbapenemase	ESBL	16S methylase	Others
p1_025943	Y	95,859					—
p2_025943	FII, FIB	75,779				*rmtB*	*aac*(*3*)*-IId*, *aac*(*6*′)*-Ib-*cr, *aadA16*, *aph*(*3*″)*-Ib*, *aph*(*6*)*-Id*, *arr-3*, *bla*_TEM-1B_, *dfrA14*, *dfrA27*, *sul1*, *sul2*, *tet*(*A*) -
p3_025943	Col (BS512)	2,088					—
pMCR1_025943	HI2, N	265,538	*mcr-1*.*1*		*bla*_CTX-M-65_		*aac*(*3*)*-IVa*, *aadA22*, *aph*(*3*′)*-Ia*, *aph*(*3*″)*-Ib*, *aph*(*6*)*-Id*, *aph*(*4*)*-Ia*, *bla*_TEM-1B_, *floR*, *lnu*(*F*), *strA*, *mphA*, *oqxA*, *oqxB*, *sul1*, *sul2*, *tet*(*A*), *tet*(*M*)
pMCR3_025943	P	50,520	*mcr-3*.*5*				—
pNDM5_025943	X3	45,275		*bla*_NDM-5_			—

^1^*bla*_CMY-2_ was located on the chromosome.

The strain was resistant to amikacin (>512 μg/ml), aztreonam (>512 μg/ml), ceftazidime (>512 μg/ml), ceftazidime-avibactam (>512/4 μg/ml), ciprofloxacin (8 μg/ml), colistin (8 μg/ml), imipenem (128 μg/ml), meropenem (128 μg/ml) and trimethoprim-sulfamethoxazole (128/2432 μg/ml), but was susceptible to aztreonam-avibactam (1/4 μg/ml) and tigecycline (0.5 μg/ml). Strain WCHEC025943 had 31 known acquired antimicrobial resistance genes mediating resistance to aminoglycosides (*aac*(*3*)*-IVa*, *aac*(*3*)*-IId*, *aac*(*6*′)*-Ib-*cr, *aadA22*, *aadA16*, *aph*(*3*′)*-Ia*, *aph*(*3*″)*-Ib*, *aph*(*6*)*-Id*, *aph*(*4*)*-Ia*, *rmtB*, *strA*), β-lactams (*bla*_CTX-M-65_, *bla*_CMY-2_, *bla*_NDM-5_, *bla*_TEM-1B_), fosfomycin (*fosA*), colistin (*mcr-1*.*1*, *mcr-3*.*5*), macrolide-lincosamide-streptogramin B (*lnu*(*F*), *mphA*), phenicol (*floR*), rifampicin (*arr-3*), quinolones (*aac*(*6*′)*-Ib-*cr, *oqxA*, *oqxB*), sulphonamides (*sul1*, *sul2*), tetracycline (*tet*(*A*), *tet*(*M*)) and trimethoprim (*dfrA14*, *dfrA27*). Compared with amikacin-susceptible strain WCHEC020123 carrying *mcr-1*.*1*, *mcr-3*.*5* and *bla*_NDM-5_^11^, strain WCHEC025943 had *rmtB*, which could explain its high-level resistance to amikacin. As a metallo-β-lactamase-encoding gene, *bla*_NDM-5_ did not confer resistance to aztreonam but the presence of *bla*_CTX-M-65_ (encoding an extended-spectrum β-lactamase) and *bla*_CMY-2_ (encoding an AmpC cephalosporinase) completed the resistance to all commercially available β-lactams including aztreonam in strain WCHEC025943.

Of note, *mcr-3*.*5* encodes three amino acid substitutions (M23V, A456E and T488I) compared with the original *mcr-3* variant on plasmid pWJ1 (GenBank accession no. KY924928). MCR-3 has been predicted to have two domains, i.e. Domain 1 (residues 1 to 172) containing 5 transmembrane α-helices and Domain 2 (residues 173 to 541), a periplasmic domain containing the putative catalytic center^5^. The amino acid substitutions of MCR-3.5 occurred in the first α-helix (M23V) of Domain 1 and in Domain 2 (A456E and T488I). However, the MIC of colistin for *E*. *coli* DH5α containing *mcr-3*.*5* was 4 μg/ml, which was identical to that for *E*. *coli* containing the original *mcr-3* variant^5^. This confirms that the amino acid substitutions of MCR-3.5 have not altered its activity against colistin as described previously^11^.

Unlike the ST206 strain WCHEC020123, strain WCHEC025943 belonged to ST410 and phylogenetic group A. ST410 (*adk-fumC-gyrB-icd-mdh-purA-recA*, 6-4-12-1-20-18-7) and ST206 (6-7-5-1-8-18-2) shared only three out of the seven alleles for MLST. ST410 *E*. *coli* has a worldwide distribution^22–24^ and a *bla*_OXA-181_-carrying ST410 *E*. *coli*, strain WCHEC14828, has been identified in the same hospital in 2014^15^. There were 109 SNPs between strain WCHEC025943 and WCHEC14828, suggesting divergence from a relatively recent common ancestor and differential plasmid acquisition and maintenance. The co-existence of *mcr-1* and *bla*_NDM-5_ has been found in *E*. *coli* isolates of various STs, such as ST156, ST446 and ST648^25,26^. Strains with resistance to both carbapenem and colistin are therefore not restricted to distinct lineages but rather are diverse in clonal background.

The *rmtB* gene in strain WCHEC025943 was carried on a 75.8-kb IncF plasmid containing an IncFII replicon (FII_47 allele) and an IncFIB replicon (a new allele). By contrast, strain WCHEC020123 did not have IncF plasmids. Like strain WCHEC020123, in strain WCHEC025943, *mcr-1*.*1*, *mcr-3*.*5* and *bla*_NDM-5_ were carried by three plasmids belonging to different replicon types (Table 1). *bla*_NDM-5_ was carried by an IncX3 plasmid, which was almost identical to the *bla*_NDM-5_-carrying IncX3 plasmid in strain WCHEC020123. *mcr-3*.*5* was carried on a 50.5-kb IncP plasmid, designated pMCR3_025943, in strain WCHEC025943. pMCR3_025943 is identical to pMCR3_020123, the *mcr-3*.*5*-carrying IncP plasmid in strain WCHEC020123^11^, except that an insertion sequence, IS1294, is absent from pMCR3_025943 but is inserted in a spacer region in pMCR3_020123. *mcr-1* was carried on a 265.5-kb plasmid (designated pMCR1_025943) containing both IncHI2 (ST3) and IncN replicons in strain WCHEC025943, which was larger than the 223.7-kb *mcr-1*-carrying IncHI2 (ST3) plasmid (pMCR1_020123) in strain WCHEC020123. The major differences between the two ST3-IncHI2 plasmids, pMCR1_025943 and pMCR1_020123, are the presence of IncN replicon and a 30-kb region containing several genes (*traN*, *traU*, *traW*) encoding conjugation in the former but absent from the latter. ST3-IncHI2 plasmids have been found increasingly as the vector of *mcr-1* and are particularly large and complex in structure with the ability to acquire multiple antimicrobial resistance genes and additional plasmid replicons^27–29^.

**Figure 1:**
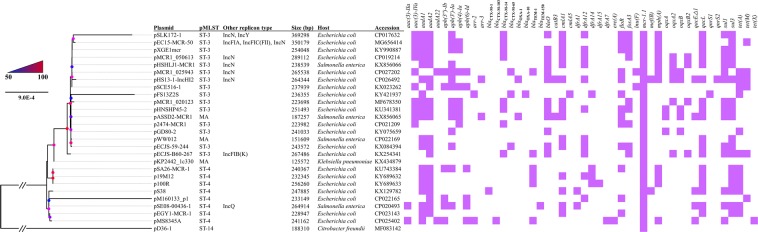
Phylogenetic analysis of *mcr-1*-carrying IncHI2 plasmids. Plasmid name, sequence type, replicon other than IncHI2, host species, plasmid size, GenBank accession no. and antimicrobial resistance genes carried are shown. Bootstrap values are indicated by dots with different degrees of colour.

**Figure 2:**
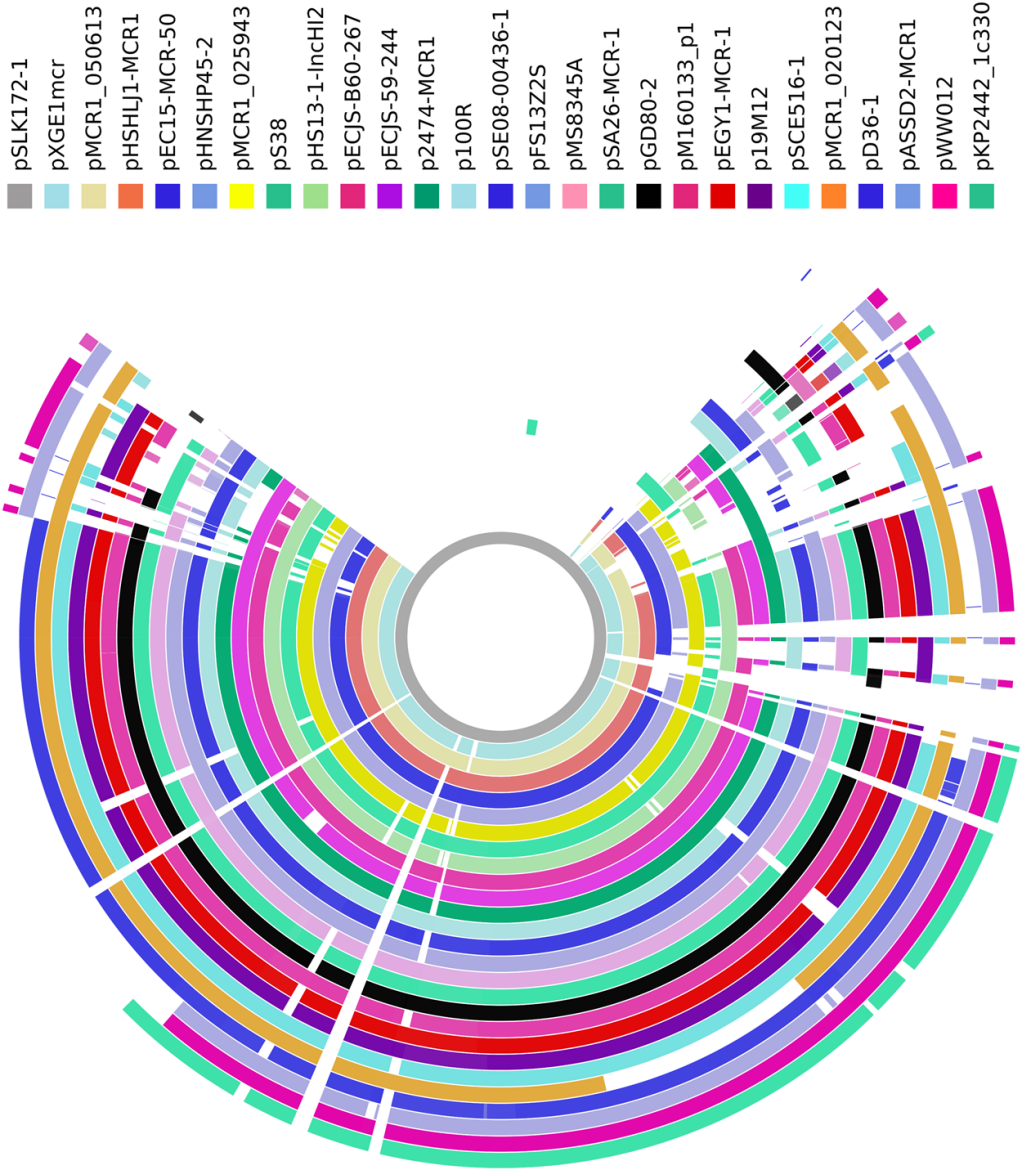
Alignment of *mcr-1*-carrying IncHI2 plasmids. Alignment was performed using BRIG^30^. pSLK172-1 (GenBank accession no. CP017632) was used as the reference due to the fact that it is the largest (369,298 bp) *mcr-1*-carrying IncHI2 plasmid and contains additional IncN and IncY replicons. GenBank accession no. of these plasmids are shown in Fig. 1.

*mcr-1*-carrying IncHI2 plasmids were mostly found in *E*. *coli* and were also present in several other species of the *Enterobacteriaceae* (Fig. 1). A few IncHI2 plasmids also contain additional replicons, among which IncN replicon was the most common (Fig. 1). These plasmids were large in size (125,572 to 256,620 bp for plasmids containing IncHI2 replicons alone and 238,539 to 369,298 bp for those containing additional replicons) and commonly carried multiple antimicrobial resistance genes (Fig. 1). Most of these plasmids belong to ST3 (n = 15) or ST4 (n = 8), while one belongs to ST14 and the sequence type is not assigned to three plasmids due to the absence of an allele for IncHI2 pMLST. This suggests that several types of IncHI2 plasmids could mediate the transfer of *mcr-1* and ST3-IncHI2 is the most common type (Fig. 1). These plasmids were also aligned against pSLK172-1 (GenBank accession no. CP017632), the largest (369,298 bp) *mcr-1*-carrying IncHI2 plasmid, using BRIG^30^. This revealed that *mcr-1*-carrying IncHI2 plasmids are complex and highly variable in structure (Fig. 2).

In strain WCHEC025943, the four plasmids carrying *mcr-1*.*1*, *mcr-3*.*5*, *bla*_NDM-5_ or *rmtB* were all self-transmissible at a 10^−3^, 10^−4^, 10^−3^ and 10^−4^ frequency, respectively. Alarmingly, the four plasmids could be transferred together to a single transconjugant at a 10^−6^ frequency. This suggests that carbapenem resistance, colistin resistance and aminoglycoside resistance can be transferred together even when their encoding genes are located on separate plasmids.

## Conclusion

The above findings suggest that carbapenem resistance, colistin resistance and high-level aminoglycoside resistance can be transferred together even when their encoding genes are not located on the same plasmid. The co-transfer of multiple clinically-important antimicrobial resistance represents a particular challenge for clinical treatment and infection control in healthcare settings, which warrant more surveillance and further studies to explore counter measures. Isolates with resistance to both carbapenem and colistin are not restricted to a given sequence type but rather are diverse in clonal background. *mcr-1*-carrying IncHI2 plasmids belonged to several sequence types with ST3 and ST4 being predominant.
